# Differenzierte Interpretation gesundheitsökonomischer Kostenkalkulationsmodelle: Ergänzung patientenbezogener Pflegebedarfe und Personalbemessung

**DOI:** 10.1007/s00113-026-01755-2

**Published:** 2026-09-01

**Authors:** Katja Hierl, Laura Schörner, Volker Alt

**Affiliations:** 1 Klinik und Poliklinik für Unfallchirurgie, Universitätsklinikum Regensburg, Franz-Josef-Strauss-Allee 11, 93053 Regensburg, Deutschland; 2 Stabsstelle Zentralcontrolling, Personalcontrolling, Universitätsklinikum Regensburg, Regensburg, Deutschland

## Erwiderung

Zum Leserbrief von Müller-Wolff T, Hermes C, Krüger L et al (2026) Personalkostenkalkulation und patientenbezogener Pflegebedarf – zwei Perspektiven einer bedarfsgerechten Personalplanung. Unfallchirurgie. https://doi.org/10.1007/s00113-026-01749-0.

## Originalbeitrag

Hierl K, Schörner L, Alt V (2026) Was kosten eine OP-Minute sowie ein Tag auf der Intensiv- und Peripherstation? Modell zur Personalkostenkalkulation von Ärztlichem Dienst und Pflegedienst in einer universitären Unfallchirurgie. Unfallchirurgie 129:111–122. https://doi.org/10.1007/s00113-025-01644-0.

Vielen Dank für Ihren Leserbrief und die konstruktiven Anmerkungen zu unserem Beitrag. Wir freuen uns, dass Sie unseren Artikel aufmerksam und mit Interesse gelesen haben und das Modell zur Personalkostenkalkulation als förderlichen Ansatz bei der ökonomischen Betrachtung der Gesundheitsversorgung und Diskussion um Kostenstrukturen im stationären Sektor einschätzen.

Gerne möchten wir auf die von Ihnen angesprochenen Punkte eingehen, die einen wertvollen Impuls zur Differenzierung und zur Weiterentwicklung modellhafter Kostenkalkulationen im Krankenhauswesen darstellen.

Wir stimmen zu, dass der reine Modellcharakter unserer Berechnungen nicht primär dafür geeignet ist, den tatsächlichen, multifaktoriell bedingten Pflegeaufwand bei der Patientenversorgung zu bestimmen. Wie zutreffend von Ihnen angemerkt wurde, sollten hierfür spezifische Methoden zu Personalbemessung und Pflegeaufwandserfassung in die Personalkostenkalkulationen einbezogen werden, um dem klinischen Alltag mit entsprechendem, häufig sehr hohem pflegerischen Versorgungsbedarf der Patienten gerecht zu werden.

Mit der Entwicklung unseres Modells sollte hingegen in erster Linie eine Möglichkeit zur zweckmäßigen Kostenkalkulation für eine definierte pflegerische und ärztliche Personalbesetzung im OP sowie auf der Intensivstation und Peripherstation aufgezeigt werden. Da dieses, wie in unserem Beitrag betont, keine universelle Gültigkeit besitzt, sondern auf unterschiedliche Personalvorhaltungen sowie Organisationsabläufe und Prozessstrukturen übertragbar ist, könnte sich das dargestellte Modell durchaus zur Erfassung der personellen Vorhaltekosten in den Kliniken eignen.

Wir sind ebenfalls der Meinung, dass für eine angemessene Abbildung sowohl der ökonomischen als auch der versorgungsbezogenen Gegebenheiten im stationären Sektor zukünftige Kostenkalkulationsmodelle den tatsächlichen patientenbezogenen Pflegeaufwand mehr berücksichtigen sollten. In Anbetracht der finanziellen Herausforderungen für die Krankenhäuser könnte dies zu einer transparenten und nachvollziehbaren Darstellung der Personalkosten beitragen.

Herzlichen Dank nochmals für die Darlegung Ihrer Sichtweise und die wertvollen Anregungen. Diese sind sehr hilfreich, um die gesundheitsökonomische Auseinandersetzung mit den Kostenstrukturen im Krankenhauswesen aus verschiedenen Blickwinkeln zu beleuchten.

## Data Availability

Alle dieser Arbeit zugrunde liegenden Daten sind in diesem Artikel enthalten.

